# Supplementary material for: Molecular motion and tridimensional nanoscale localization of kindlin control integrin activation in focal adhesions
Source: Nat Commun. 2021 May 25;12:3104. doi: 10.1038/s41467-021-23372-w (PMC8149821; doi:10.1038/s41467-021-23372-w)
Supplement: Supplementary file 1 — Supplementary Information [file 41467_2021_23372_MOESM1_ESM.pdf]

SUPPLEMENTARY INFORMATION

Molecular motion and tridimensional nanoscale localization of kindlin control integrin activation in focal adhesions

Thomas Orré[1], Adrien Joly[1§], Zeynep Karatas[1§], Birgit Kastberger[2], Clément Cabriel[3], Ralph T. Böttcher[4], Sandrine Lévêque-Fort[3], Jean-Baptiste Sibarita[1], Reinhard Fässler[4], Bernhard Wehrle-Haller[2], Olivier Rossier[1,*], Grégory Giannone[1,*]

1. Univ. Bordeaux, CNRS, Interdisciplinary Institute for Neuroscience, IINS, UMR 5297, F-33000 Bordeaux, France

2. Dept. of Cell Physiology and Metabolism, Centre Médical Universitaire, 1211 Geneva 4, Switzerland

3. Institut des Sciences Moléculaires d'Orsay, CNRS UMR8214, Univ. Paris-Sud, Université Paris Saclay, 91405, Orsay Cedex F91405, France

4. Max Planck Institute of Biochemistry, Martinsried, Germany

These authors contributed equally: Adrien Joly, Zeynep Karatas. These authors jointly supervised this work: Olivier Rossier, Grégory Giannone. Correspondence and requests for materials should be addressed to Olivier Rossier (olivier.rossier@u-bordeaux.fr) and Grégory Giannone (gregory.giannone@u-bordeaux.fr).

This part includes

Supplementary Figures 1 to 9

Supplementary Tables 1 to 7

Other Supplementary Information for this manuscript includes the following:

Supplementary Movies 1 to 3

SUPPLEMENTARY FIGURES

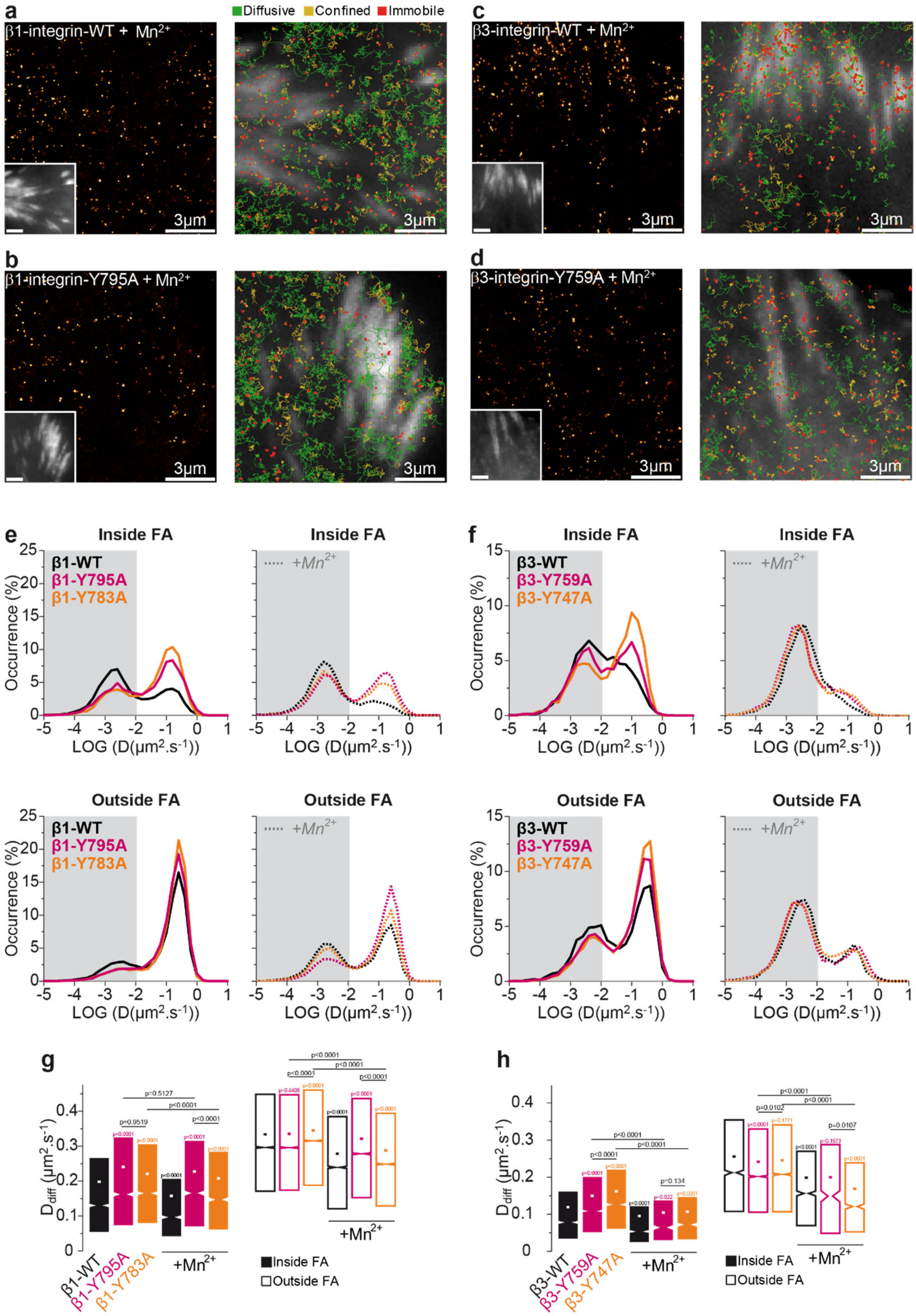

Supplementary figure 1. Kindlin is required for Mn^{2+}-induced β1-integrin immobilization. (**a**-**d**) Left: Super-resolution intensity images of β1-WT-mEos2 (a), β1-Y95A-mEos2 (b), β3-WT-mEos2 (c), β3-Y759A-mEos2 (d) in Mn^{2+}-stimulated MEFs obtained from a sptPALM sequence (50 Hz, >80 s). Inset: low resolution image of GFP-paxillin, which was co-expressed for FAs labelling (scale bar: 3 µm). Right: color-coded trajectories overlaid on FAs labelled by GFP-paxillin (greyscale) show the diffusion modes: free diffusion (green), confined diffusion (yellow) and immobilization (red). Micrographs (a-d) are representative images of three independent experiments unless indicated: β1-WT-mEos2 with Mn^{2+} (15 cells); β1-Y795A-mEos2 with Mn^{2+} (22 cells); β3-mEos2 with Mn^{2+} (13 cells); β3-Y759A-mEos2 with Mn^{2+} (11 cells, 2 ind. exp.) (**e**) Distributions of the diffusion coefficient D, computed from the trajectories obtained inside (top) and outside (bottom) FAs with β1-WT-mEos2 (black), β1-Y795A-mEos2 (pink), β1-Y783A-mEos2 (orange) in MEFs (left, full lines) and in Mn^{2+}-stimulated MEFs (right, dashed lines), are shown in a logarithmic scale. The grey area including D values inferior to 0.011 $µm^2.s^{-1}$ corresponds to immobilized proteins. Values represent the average of the distributions obtained from different cells. (**f**) Same as **e**, but with β3-WT-mEos2 (black), β3-Y759A-mEos2 (pink), β3-Y747A-mEos2 (orange). (**g**) Box plots displaying the median (notch) and mean (square) ± percentile (25–75%) of diffusion coefficients corresponding to the free diffusion trajectories of β1-WT-mEos2 (black), β1-Y795A-mEos2 (pink), β1-Y783A-mEos2 (orange) inside (left) and outside (right) FAs. (**h**) Same as **g**, but with β3-WT-mEos2 (black), β3-Y759A-mEos2 (pink), β3-Y747A-mEos2 (orange). Results for β1-WT-mEos2 without (16 cells) and with Mn^{2+} (15 cells); β1-Y795A-mEos2 (22 cells) and with Mn^{2+} (22 cells); β1-Y783A-mEos2 without (16 cells) and with Mn^{2+} (20 cells); β3-mEos2 without (16 cells) and with Mn^{2+} (13 cells); β3-Y759A-mEos2 without (20 cells) and with Mn^{2+} (11 cells, 2 ind. exp.) and β3-Y747A-mEos2 without (16 cells) and with Mn^{2+} (10 cells, 2 ind. exp.) correspond to pooled data from three independent experiments unless indicated. Where indicated, statistical significance was obtained using two-tailed, non-parametric Mann–Whitney rank sum test. Inside and outside FAs, the different conditions without Mn^{2+} were compared to the corresponding β-integrin-WT condition; with Mn^{2+}, each given condition was compared to the value obtained without Mn^{2+}. Otherwise, a black line indicates which conditions were compared. The exact P values are indicated on the figure except when $P < 0.0001$. Source data are provided as a Source Data file.

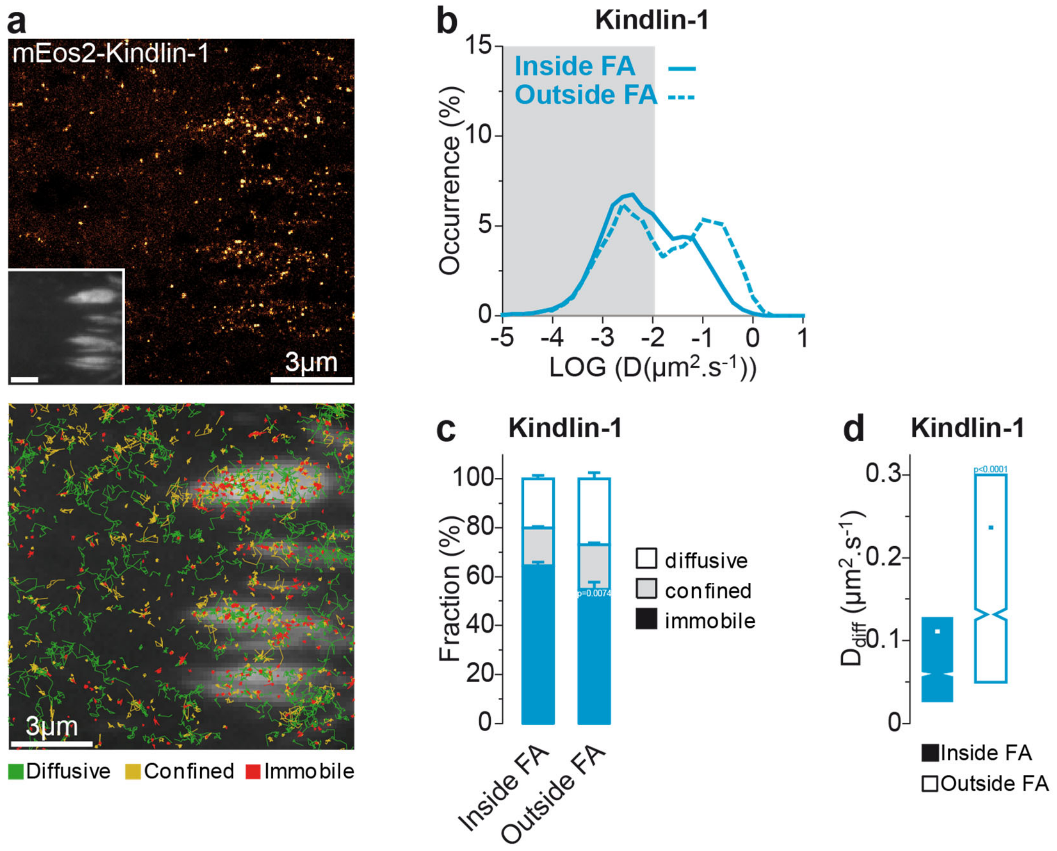

Supplementary figure 2. Kindlin-1 undergoes lateral free diffusion along the plasma membrane. (**a**) Top: Super-resolution PALM intensity images of mEos2-kindlin-1 in a MEF obtained from a sptPALM sequence (50 Hz, >80 s). Inset: low resolution image of GFP-paxillin, which was co-expressed for FAs labelling (scale bar: 3 µm). Bottom: color-coded trajectories overlaid on FAs labelled by GFP-paxillin (greyscale) show the diffusion modes: free diffusion (green), confined diffusion (yellow) and immobilisation (red). Micrograph of mEos2-kindlin-1 is a representative image of three independent experiments (25 cells). (**b**) Distributions of the diffusion coefficient D computed from the trajectories of mEos2-kindlin-1 obtained inside (full line) and outside (dashed line) FAs are shown in a logarithmic scale. The grey area including D values inferior to 0.011 $\mu m^2.s^{-1}$ corresponds to immobilized proteins. Values represent the average of the distributions obtained from different cells. (**c**) Fraction of mEos2-kindlin-1 undergoing free diffusion, confined diffusion or immobilisation inside (left) and outside (right) FAs. Values represent the average of the fractions obtained from different cells (error bars: SEM). (**d**) Box plots displaying the median (notch) and mean (square) ± percentile (25–75%) of diffusion coefficients corresponding to the free diffusion trajectories of mEos2-kindlin-1 inside (left) and outside (right) FAs. Results correspond to pooled data from three independent experiments (25 cells). Where indicated, statistical significance was obtained using two-tailed, non-parametric Mann–Whitney rank sum test. The exact P values are indicated on the figure except when P < 0.0001. Source Data are provided as a Source Data file.

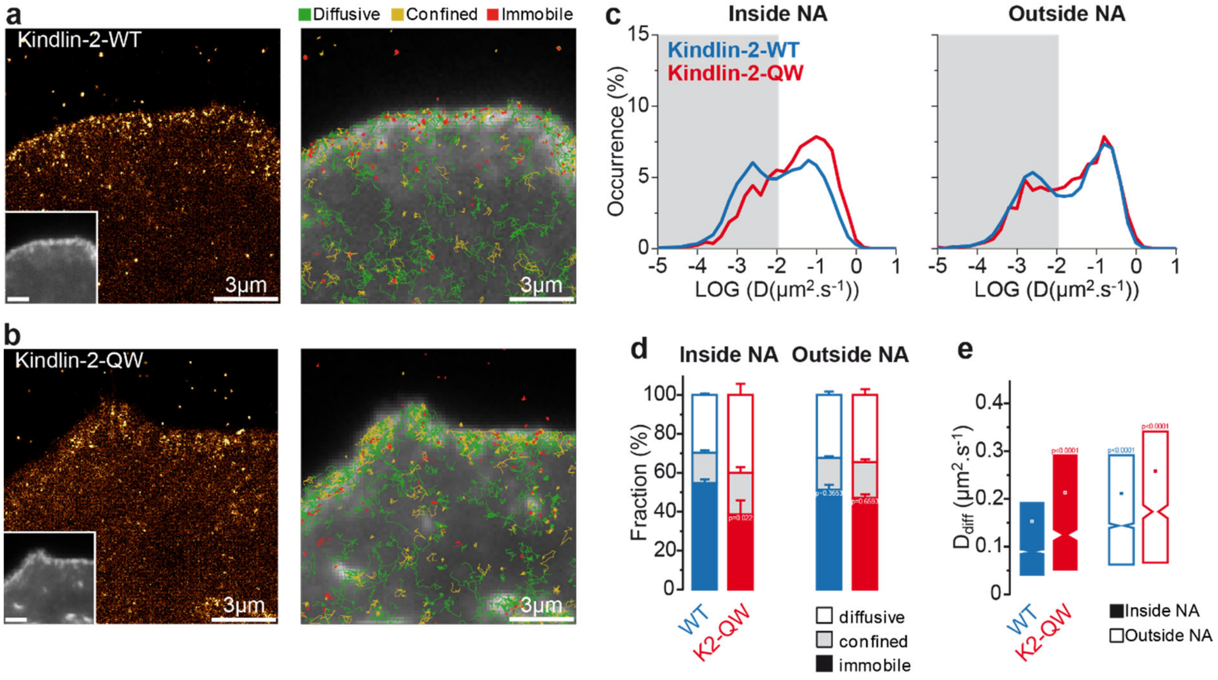

Supplementary figure 3. Kindlin-2 also displays membrane free diffusion both inside and outside NAs in spreading MEFs. (**a**-**b**) Left: Super-resolution PALM intensity images of mEos2-kindlin-2-WT (a) and mEos2-kindlin-2-QW614/615AA (b) in spreading MEFs obtained from a sptPALM sequence (50 Hz, >30 s). Inset: low resolution image of GFP-paxillin, which was co-expressed for NAs labelling (scale bar: 3 µm). Right: color-coded trajectories overlaid on NAs labelled by GFP-paxillin (greyscale) show the diffusion modes: free diffusion (green), confined diffusion (yellow) and immobilization (red). Micrographs for mEos2-kindlin-2-WT and mEos2-kindlin-2-QW614/615AA are representative images of two independent experiments (mEos2-kindlin-2-WT (11 cells) and mEos2-kindlin-2-QW614/615AA (3 cells). (**c**) Distributions of the diffusion coefficient D computed from the trajectories of mEos2-kindlin-2-WT (blue), mEos2-kindlin-2-QW614/615AA (red) obtained inside (left) and outside NAs (right), are shown in a logarithmic scale. The grey area including D values inferior to 0.011 $\mu m^2.s^{-1}$ corresponds to immobilized proteins. Values represent the average of the distributions obtained from different cells. (**d**) Fraction of proteins undergoing free diffusion, confined diffusion or immobilization inside (left) and outside NAs (right) for mEos2-kindlin-2-WT, mEos2-kindlin-2-QW614/615AA (same color-code). Values represent the average of the fractions obtained from several cells (error bars: SEM). (**e**) Box plots displaying the median (notch) and mean (square) ± percentile (25–75%) of diffusion coefficients corresponding to the free diffusion trajectories inside (left) and outside NAs (right). Results for mEos2-kindlin-2-WT (11 cells) and mEos2-kindlin-2-QW614/615AA (3 cells) correspond to pooled data from two independent experiments. Where indicated, statistical significance was obtained using two-tailed, non-parametric Mann–Whitney rank sum test. Inside and outside FAs, the different conditions were compared to the corresponding mEos2-kindlin-2-WT condition. The exact P values are indicated on the figure except when $P < 0.0001$. Source data are provided as a Source Data file.

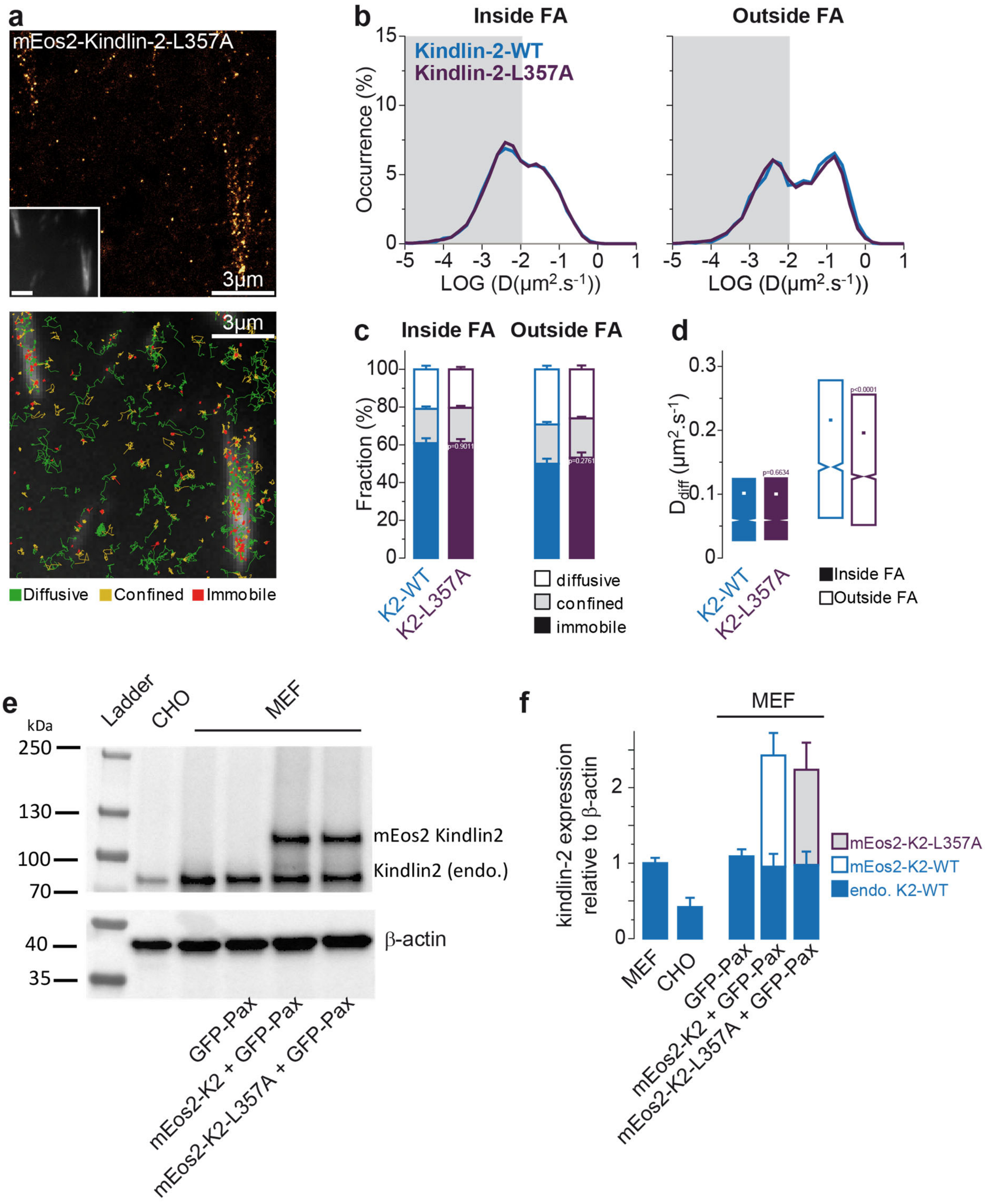

Supplementary figure 4. Kindlin-2 membrane diffusion and immobilization does not depend on the interaction with ILK. (**a**) Top: Super-resolution PALM intensity images of mEos2-kindlin-2-L357A in MEFs obtained from a sptPALM sequence (50 Hz, >80 s). Inset: low resolution image of GFP-paxillin, which was co-expressed for FAs labelling (scale bar: 3 μm). Bottom: color-coded trajectories overlaid on FAs labelled by GFP-paxillin (greyscale) show the diffusion modes: free diffusion (green), confined diffusion (yellow) and immobilization (red). Micrograph of mEos2-kindlin-2-L357A is a representative image of three independent experiments (22 cells). (**b**) Distributions of the diffusion coefficient D computed from the trajectories of mEos2-kindlin-2-WT (blue) and mEos2-kindlin-2-L357A (purple)

obtained inside (left) and outside FAs (right), are shown in a logarithmic scale. The grey area including D values inferior to 0.011 $\mu m^2.s^{-1}$ corresponds to immobilized proteins. Values represent the average of the distributions obtained from different cells. (**c**) Fraction of proteins undergoing free diffusion, confined diffusion or immobilization inside (left) and outside (right) adhesion sites. Values represent the average of the fractions obtained from different cells (error bars: SEM). (**d**) Box plots displaying the median (notch) and mean (square) ± percentile (25–75%) of diffusion coefficients corresponding to the free diffusion trajectories inside (left) and outside FAs (right). Results for mEos2-kindlin-2-WT (12 cells) and mEos2-kindlin-2-L357A (22 cells) correspond to pooled data from three independent experiments. Where indicated, statistical significance was obtained using two-tailed, non-parametric Mann–Whitney rank sum test. Inside and outside FAs, the mEos2-kindlin-2-L357A conditions were compared to the corresponding mEos2-kindlin-2-WT condition. The exact P values are indicated on the figure except when $P < 0.0001$. (**e**) Western blot using an anti-kindlin-2 antibody characterizing the expression levels of endogenous and transfected kindlin-2 protein in CHO cells (lane 1), MEFs (lane 2) and MEFs transfected with GFP-paxillin alone (lane 3) or with mEos2-tagged kindlin-2-WT (lane 4) or -L357A mutant (lane 5). β-actin is used as a loading control. (**f**) Quantification of kindlin-2 expression levels normalized to the expression levels of endogenous kindlin-2 in MEFs (mean±s.e.m., n=3) with endogenous kindlin-2 (plain blue), transfected mEos2-tagged kindlin-2-WT (hollow blue) or -L357A mutant (hollow purple). Source data are provided as a Source Data file.

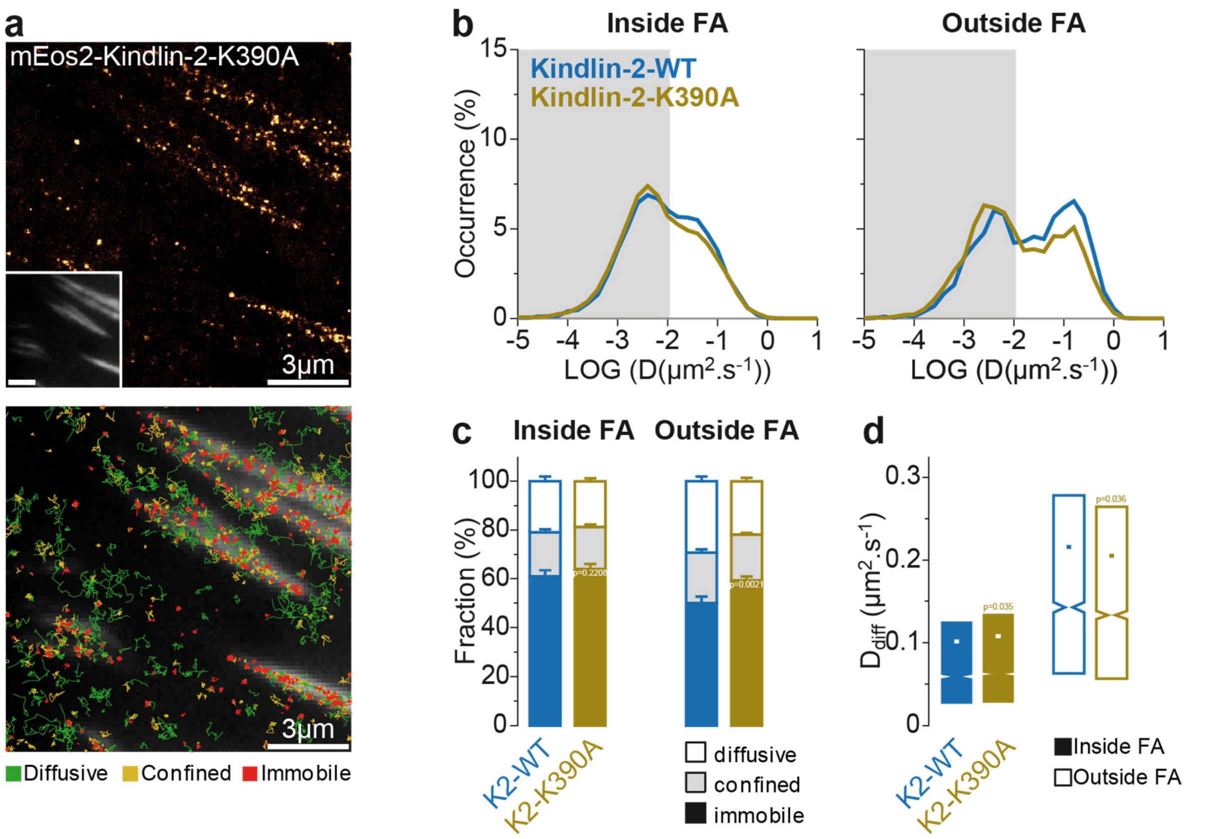

Supplementary figure 5. Disrupting the interaction of kindlin with phosphoinositides decreases kindlin membrane diffusion. (**a**) Top: Super-resolution PALM intensity images of mEos2-kindlin-2-K390A in MEFs obtained from a sptPALM sequence (50 Hz, >80 s). Inset: low resolution image of GFP-paxillin, which was co-expressed for FAs labelling (scale bar: 3 µm). Bottom: color-coded trajectories overlaid on FAs labelled by GFP-paxillin (greyscale) show the diffusion modes: free diffusion (green), confined diffusion (yellow) and immobilization (red). Micrograph of mEos2-kindlin-2-K390A is a representative image of three independent experiments (23 cells). (**b**) Distributions of the diffusion coefficient D computed from the trajectories of mEos2-kindlin-2-WT (blue) and mEos2-kindlin-2-K390A (khaki) obtained inside (left) and outside FAs (right), are shown in a logarithmic scale. The grey area including D values inferior to 0.011 $\mu m^2.s^{-1}$ corresponds to immobilized proteins. Values represent the average of the distributions obtained from different cells. (**c**) Fraction of proteins undergoing free diffusion, confined diffusion or immobilization inside (left) and outside FAs (right). Values represent the average of the fractions obtained from different cells (error bars: SEM). (**d**) Box plots displaying the median (notch) and mean (square) ± percentile (25–75%) of diffusion coefficients corresponding to the free diffusion trajectories inside (left) and outside FAs (right). Results for mEos2-kindlin-2-WT (12 cells) and mEos2-kindlin-2-K390A (23 cells) correspond to pooled data from three independent experiments. Where indicated, statistical significance was obtained using two-tailed, non-parametric Mann–Whitney rank sum test. Inside and outside FAs, the mEos2-kindlin-2-K390A conditions were compared to the corresponding mEos2-kindlin-2-WT condition. The exact P values are indicated on the figure except when P < 0.0001. Source data are provided as a Source Data file.

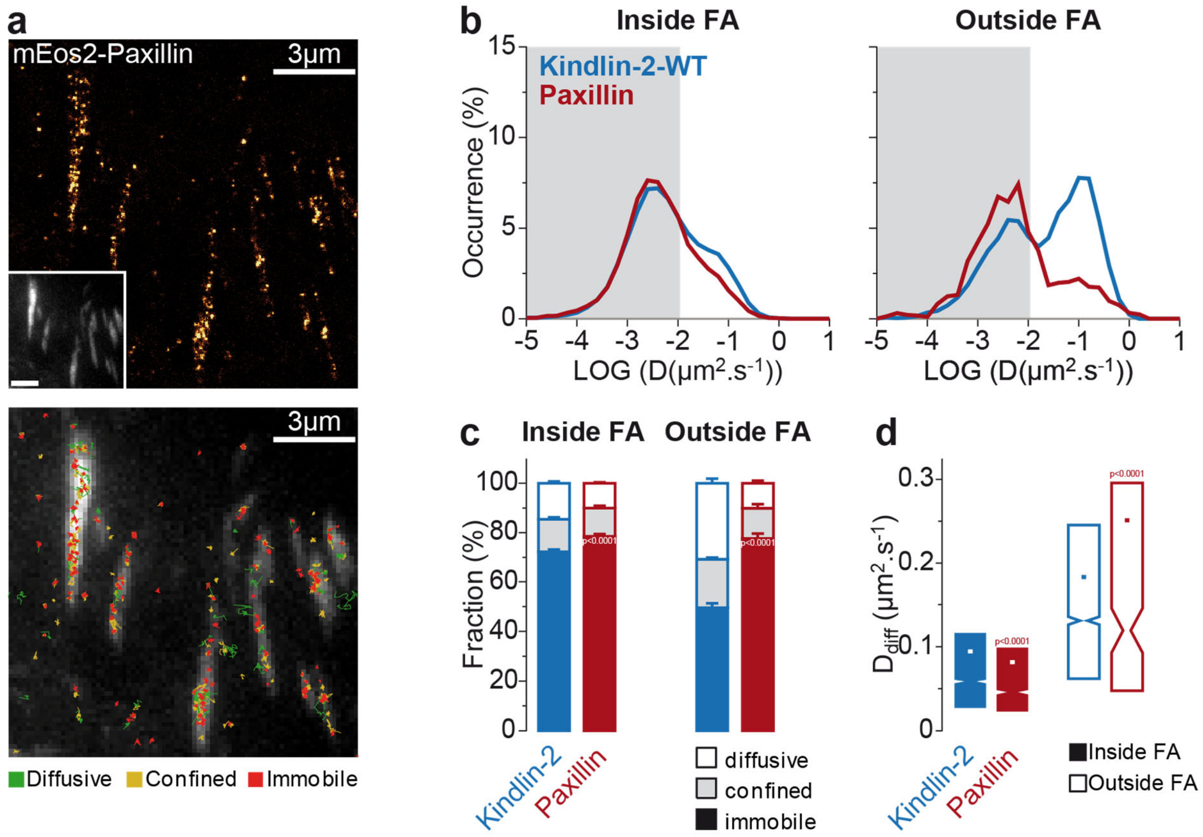

Supplementary figure 6. Kindlin-2 diffusion is not driven by paxillin, which is mostly immobile. (**a**) Top: Super-resolution PALM intensity images of mEos2-paxillin in MEFs obtained from a sptPALM sequence (50 Hz, >80 s). Inset: low resolution image of GFP-paxillin, which was co-expressed for FAs labelling (scale bar: 3 µm). Bottom: color-coded trajectories overlaid on FAs labelled by GFP-paxillin (greyscale) show the diffusion modes: free diffusion (green), confined diffusion (yellow) and immobilization (red). Micrograph of mEos2-Paxillin is a representative image of two independent experiments (12 cells). (**b**) Distributions of the diffusion coefficient D computed from the trajectories of mEos2-kindlin-2-WT (blue) and mEos2-paxillin (red) obtained inside (left) and outside FAs (right), are shown in a logarithmic scale. The grey area including D values inferior to 0.011 $\mu m^2.s^{-1}$ corresponds to immobilized proteins. Values represent the average of the distributions obtained from different cells. (**c**) Fraction of proteins undergoing free diffusion, confined diffusion or immobilization inside (left) and outside FAs (right). Values represent the average of the fractions obtained from different cells (error bars: SEM). (**d**) Box plots displaying the median (notch) and mean (square) ± percentile (25–75%) of diffusion coefficients corresponding to the free diffusion trajectories inside (left) and outside FAs (right). Results for mEos2-kindlin-2-WT (10 cells) mEos2-paxillin (12 cells) correspond to pooled data from two independent experiments. Where indicated, statistical significance was obtained using two-tailed, non-parametric Mann–Whitney rank sum test. Inside and outside FAs, the mEos2-paxillin conditions were compared to the corresponding mEos2-kindlin-2-WT condition. The exact P values are indicated on the figure except when $P < 0.0001$. Source data are provided as a Source Data file.

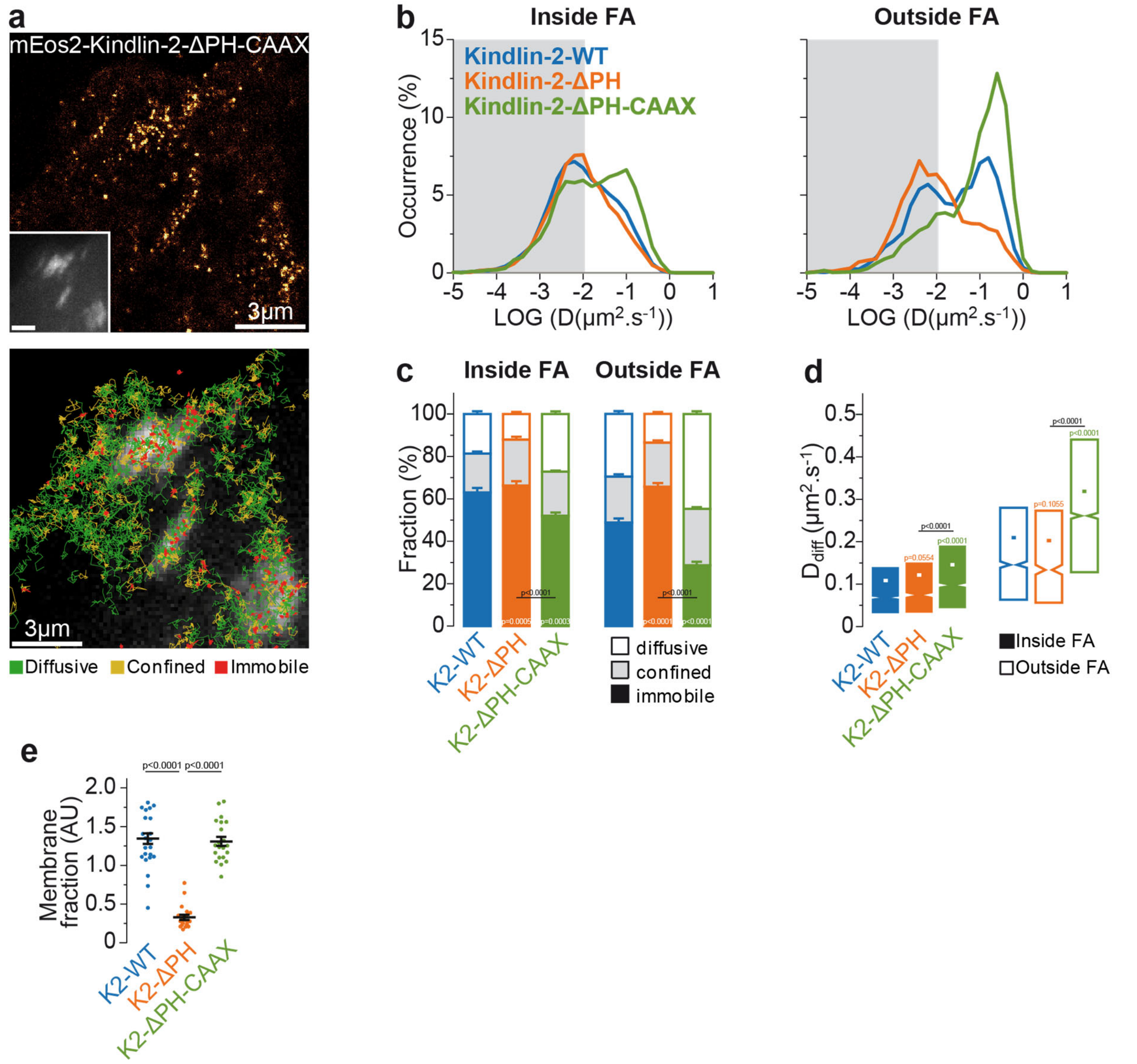

Supplementary figure 7. Adding a CAAX sequence in kindlin-2-ΔPH induces high membrane recruitment and diffusion. (**a**) Top: Super-resolution PALM intensity images of mEos2-kindlin-2-ΔPH-CAAX in MEFs, obtained from a sptPALM sequence (50 Hz, >80 s). Inset: low resolution image of GFP-paxillin, which was co-expressed for FAs labelling (scale bar: 3 µm). Bottom: color-coded trajectories overlaid on FAs labelled by GFP-paxillin (greyscale) show the diffusion modes: free diffusion (green), confined diffusion (yellow) and immobilization (red). Micrograph of mEos2-kindlin-2-ΔPH-CAAX is a representative image of three independent experiments (18 cells). (**b**) Distributions of the diffusion coefficient D computed from the trajectories of mEos2-kindlin-2-WT (blue), mEos2-kindlin-2-ΔPH (orange) and mEos2-kindlin-2-ΔPH-CAAX (green) obtained inside (left) and outside FAs (right), are shown in a logarithmic scale. The grey area including D values inferior to 0.011 $\mu m^2.s^{-1}$ corresponds to immobilized proteins. Values represent the average of the distributions obtained from different cells. (**c**) Fraction of proteins undergoing free diffusion, confined diffusion or immobilization inside (left) and outside FAs (right). Values represent the average of the fractions obtained from different cells (error bars: SEM). (**d**) Box plots displaying the median (notch) and mean (square) ± percentile (25–75%) of diffusion coefficients corresponding to the free diffusion trajectories inside (left) and outside FAs (right). (**e**) Fraction of proteins recruited at the membrane in kindlin-1, kindlin-2 knock-

out cells (Kind^{Ko}) quantified by the ratio of the membrane-level fluorescence signal (TIRF) to the total fluorescence signal of the cell (epifluorescence). Fluorescence was measured in the red channel after photoconversion of the mEos2-genetically-coupled indicated proteins. Black bars represent mean and SEM. For sptPALM data, results for mEos2-kindlin-2-WT (15 cells), mEos2-kindlin-2-ΔPH (28 cells) and mEos2-kindlin-2-ΔPH-CAAX (18 cells) correspond to pooled data from three independent experiments. For membrane fraction data in Kind^{Ko}, results for mEos2-kindlin-2-WT (21 cells), mEos2-kindlin-2-ΔPH (20 cells) and mEos2-kindlin-2-ΔPH-CAAX (21 cells) come from a same experiment. Where indicated, statistical significance was obtained using two-tailed, non-parametric Mann–Whitney rank sum test. Inside and outside FAs, the different conditions were compared to the corresponding mEos2-kindlin-2-WT condition. The exact P values are indicated on the figure except when $P < 0.0001$. Source data are provided as a Source Data file.

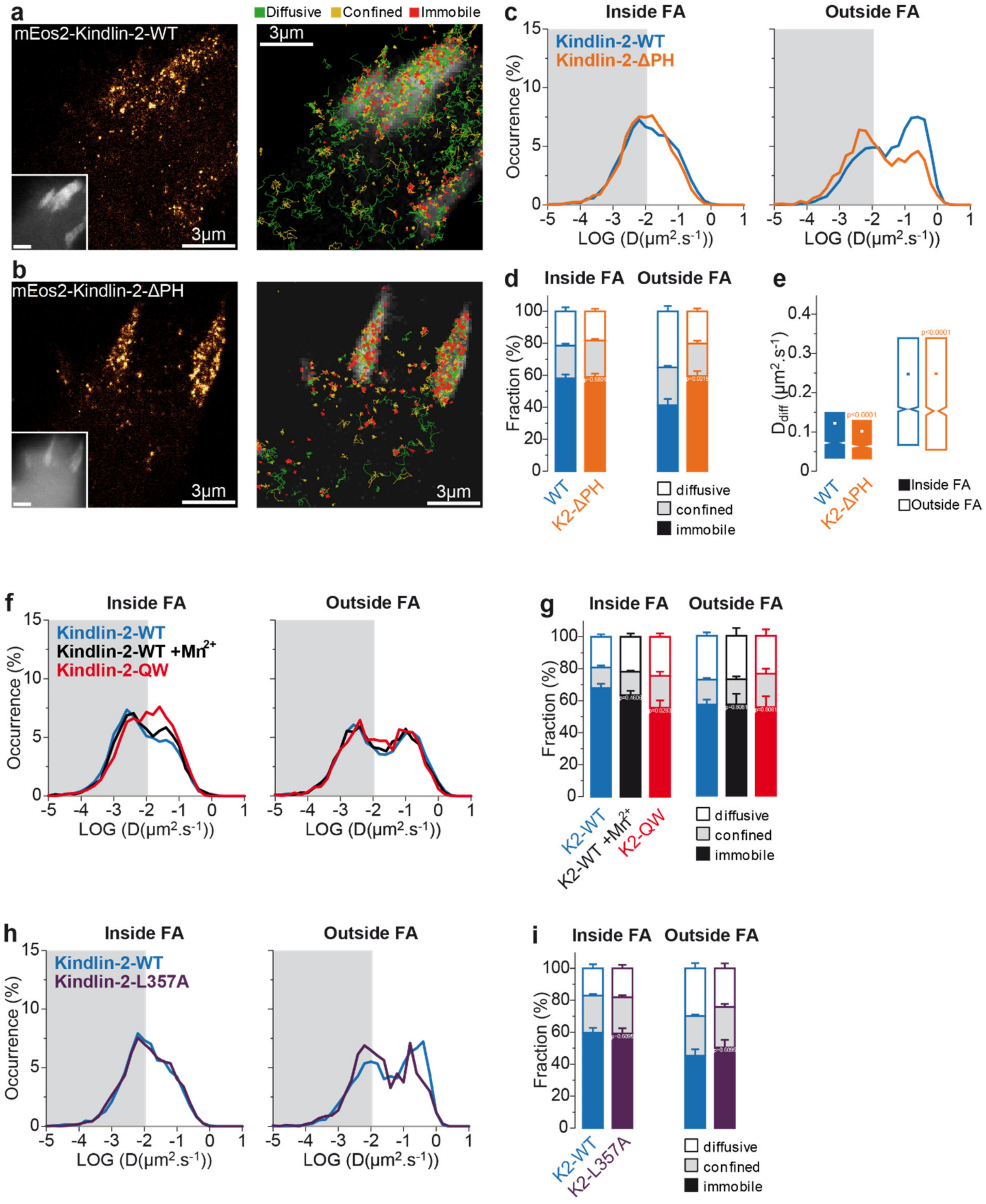

Supplementary figure 8. MEF and Kind^{Ko} (kindlin-1, kindlin-2 knock out) cells give similar sptPALM results. All data shown were obtained in Kind^{Ko} cells. (**a**-**b**) Left: Super-resolution intensity images of mEos2-kindlin-2-WT (a) and mEos2-kindlin-2-ΔPH (b), obtained from a sptPALM sequence (50 Hz, >80 s). Inset: low resolution image of GFP-paxillin, which was co-expressed for FAs labelling (scale bar: 3 µm). Right: color-coded trajectories overlaid on FAs labelled by GFP-paxillin (greyscale) show the diffusion modes: free diffusion (green), confined diffusion (yellow) and immobilization (red). Micrographs for mEos2-kindlin-2-WT and mEos2-kindlin-2-ΔPH are representative images of two independent experiments (mEos2-kindlin-2-WT (11 cells) and mEos2-kindlin-2-ΔPH (18 cells)). (**c**)

Distributions of the diffusion coefficient D computed from the trajectories of mEos2-kindlin-2-WT (blue) and mEos2-kindlin-2-ΔPH (orange) obtained inside (left) and outside FAs (right), are shown in a logarithmic scale. The grey area including D values inferior to 0.011 $\mu m^2.s^{-1}$ corresponds to immobilized proteins. Values represent the average of the distributions obtained from different cells. (**d**) Fraction of proteins undergoing free diffusion, confined diffusion or immobilization inside (left) and outside FAs (right). Values represent the average of the fractions obtained from different cells (error bars: SEM). (**e**) Box plots displaying the median (notch) and mean (square) ± percentile (25–75%) of diffusion coefficients corresponding to the free diffusion trajectories inside (left) and outside FAs (right). (**f**-**g**) Same as in **c**-**d**, but for mEos2-kindlin-2-WT (blue), mEos2-kindlin-2-WT in Mn^{2+}-stimulated cells (black) and mEos2-kindlin-2-QW614/615AA (red). Values represent the average of the fractions obtained from different cells (error bars: SEM). (**h**-**i**) Same as in **c**-**d**, but for mEos2-kindlin-2-WT (blue) and mEos2-kindlin-2-L357A (purple). Values represent the average of the fractions obtained from different cells (error bars: SEM). Results in (a-e) for mEos2-kindlin-2-WT (11 cells) and mEos2-kindlin-2-ΔPH (18 cells) correspond to pooled data from two independent experiments. (f,g): Results for mEos2-kindlin-2-WT (8 cells), mEos2-kindlin-2-WT +Mn^{2+} (4 cells), mEos2-kindlin-2-QW614/615AA (4 cells) come from a single experiment. (h,i): Results for mEos2-kindlin-2-WT (6 cells), and mEos2-kindlin-2-L357A (4 cells) come from a single experiment. Where indicated, statistical significance was obtained using two-tailed, non-parametric Mann–Whitney rank sum test. Inside and outside FAs, the different conditions were compared to the corresponding mEos2-kindlin-2-WT condition. The exact P values are indicated on the figure except when $P < 0.0001$. Source data are provided as a Source Data file.

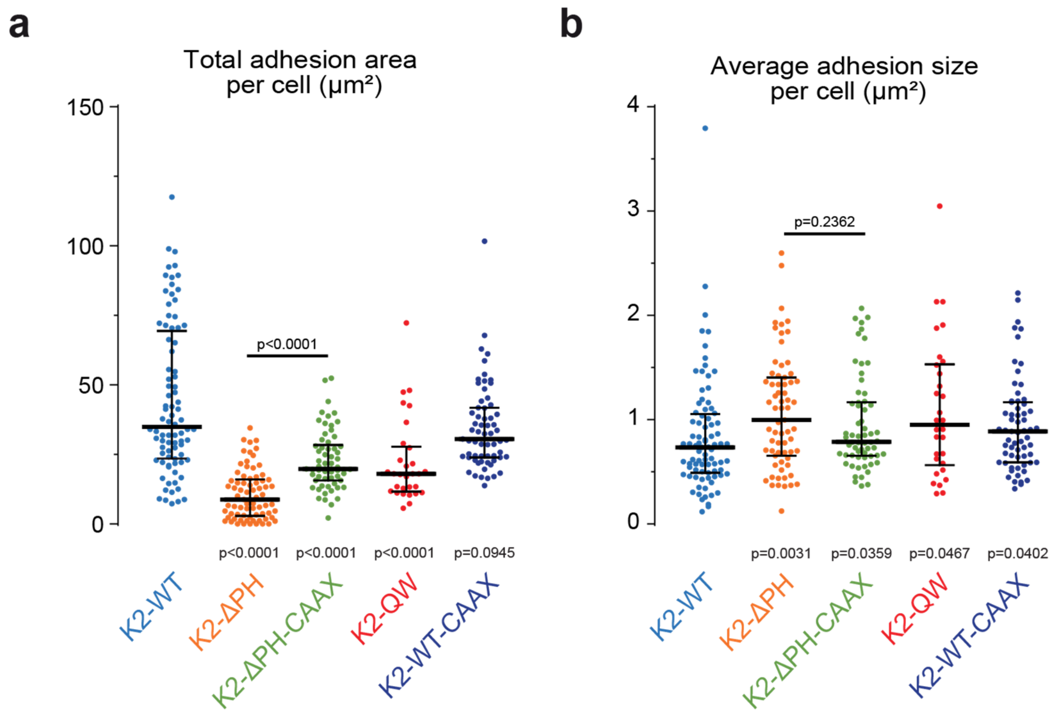

Supplementary figure 9. PH-domain-mediated membrane recruitment and diffusion of kindlin-2 is crucial during mature FA formation, but not for FA size. Quantification of total area of FAs per cell (**a**) and mean FA area (**b**) of Kind^{Ko} cells (4h after seeding on fibronectin) re-expressing for 2 days mEos2-kindlin-2-WT (light blue, 84 cells), mEos2-kindlin-2-ΔPH (orange, 74 cells), mEos2-kindlin-2-ΔPH-CAAX (green, 62 cells), mEos2-kindlin-2-QW614/615AA (red, 30 cells) or mEos2-kindlin-2-WT-CAAX (dark blue, 63 cells). Each point in the distribution represents the value obtained from a single cell. FAs were drawn manually and cell boundaries were determined by manually setting a threshold on the pixel intensity values using the TIRF GFP-paxillin images as shown in Fig. 7c. Black bars represent medians and interquartile ranges. The results correspond to pooled data from three independent experiments. Where indicated, statistical significance was obtained using two-tailed, non-parametric Mann–Whitney rank sum test. The exact P values are indicated on the figure except when P < 0.0001. Source data are provided as a Source Data file.

SUPPLEMENTARY TABLES

Table S1. Results of sptPALM experiments (FAs: Focal adhesions; NAs: nascent adhesions).

	β1-integrin-WT	β1-integrin-Y795A	β1-integrin-Y783A	β1-integrin-WT + Mn2+	β1-integrin-Y795A +Mn2+
cells	17	22	16	15	22
trajectories inside FAs	25270	12531	11606	20775	11243
trajectories outside FAs	64523	55527	74583	56565	66450
	**Mean ± SEM (median)**	**Mean ± SEM (median)**	**Mean ± SEM (median)**	**Mean ± SEM (median)**	**Mean ± SEM (median)**
immobile inside FAs (%)	71.7 ± 1.5 (72.6)	47.9 ± 2.9 (47.7)	41.2 ± 1.7 (41.2)	83.4 ± 1.1 (83.9)	60.3 ± 2.1 (60.3)
confined inside FAs (%)	11.2 ± 0.5 (11.3)	20.5 ± 1.1 (20.8)	22.6 ± 0.8 (21.8)	7.1 ± 0.7 (7.1)	14.8 ± 0.8 (15.3)
diffusive inside FAs (%)	17.1 ± 1.2 (17.4)	31.6 ± 2 (30.9)	36.3 ± 1 (37.1)	9.5 ± 0.6 (9.6)	24.9 ± 1.6 (24.1)
immobile outside FAs (%)	31.6 ± 2.3 (31.4)	19.4 ± 1.1 (19.8)	19.1 ± 1.2 (18.3)	58.7 ± 2.6 (57.3)	36.8 ± 2.5 (35.9)
confined outside FAs (%)	20 ± 0.6 (20.3)	25.5 ± 0.5 (25.7)	22.8 ± 0.4 (23.1)	13.4 ± 1 (13.6)	21.1 ± 1.1 (20.8)
diffusive outside FAs (%)	48.3 ± 2 (48)	55.1 ± 1.1 (55)	58.1 ± 1.2 (58.1)	27.9 ± 2 (29.1)	42 ± 1.8 (41.9)
D_{diff} inside x10^3 ($\mu m^2.s^{-1}$)	195.9 ± 3.4 (127.5)	237.5 ± 4 (159.3)	217.7 ± 3 (162.1)	154.8 ± 3.9 (93.7)	224.4 ± 4.2 (162.8)
D_{diff} outside x10^3 ($\mu m^2.s^{-1}$)	330.7 ± 1.3 (293.6)	332.5 ± 1.3 (293.1)	342 ± 1 (312.4)	275.4 ± 1.7 (236.5)	318.7 ± 1.4 (275.5)

	β1-integrin-Y783A +Mn2+	β3-integrin-WT	β3-integrin-Y759A	β3-integrin-Y747A	β3-integrin-WT +Mn2+
cells	20	16	20	16	13
trajectories inside FA	13615	11347	18438	10624	16487
trajectories outside FA	82547	9355	34797	28579	78006
	**Mean ± SEM (median)**	**Mean ± SEM (median)**	**Mean ± SEM (median)**	**Mean ± SEM (median)**	**Mean ± SEM (median)**
immobile inside FA (%)	68 ± 2.1 (69.7)	61.7 ± 1.5 (62.2)	57 ± 1.5 (58.1)	46.1 ± 2.5 (45.7)	82.7 ± 1.5 (85.9)
confined inside FA (%)	12.1 ± 0.8 (12.3)	20.2 ± 1.1 (20.1)	18.4 ± 0.8 (17.3)	19.5 ± 0.7 (19.5)	11 ± 0.9 (9.2)
diffusive inside FA (%)	19.9 ± 1.4 (18.5)	18.1 ± 1.4 (17.6)	24.6 ± 1.3 (24.3)	34.4 ± 2.2 (34.4)	6.3 ± 0.8 (4.9)
immobile outside FA (%)	51.5 ± 2.3 (51.4)	49 ± 2.9 (49)	41 ± 2.5 (40.6)	36.3 ± 2.3 (37.2)	77 ± 1.7 (78.9)
confined outside FA (%)	15.1 ± 0.6 (15)	19.3 ± 1.2 (19.8)	20.8 ± 0.9 (21.7)	20.1 ± 0.8 (19.9)	11.3 ± 0.5 (11.1)
diffusive outside FA (%)	33.4 ± 1.8 (32.6)	31.6 ± 2.4 (30.3)	38.2 ± 1.9 (37.9)	43.7 ± 2 (42.6)	11.7 ± 1.4 (10)

D_{diff} inside x10^3 (µm^2.s^{-1})	205.2 ± 4 (144.3)	119.6 ± 2.7 (78.5)	150.2 ± 2.1 (109.1)	162.5 ± 2.4 (126.5)	95.6 ± 3.2 (53.6)
D_{diff} outside x10^3 (µm^2.s^{-1})	284.9 ± 1.3 (245.8)	257 ± 3.6 (213.1)	242.5 ± 1.6 (202.5)	246.6 ± 1.7 (208.6)	199.4 ± 3.7 (156.7)

	β3-integrin-Y759A +Mn2+	β3-integrin-Y747A +Mn2+	Kindlin-2-WT (Fig 2)	Talin-1	Kindlin-1
cells	11	10	13	8	25
trajectories inside FA	15660	12783	15475	18159	17305
trajectories outside FA	18685	24649	9792	7940	17940
	**Mean ± SEM (median)**	**Mean ± SEM (median)**	**Mean ± SEM (median)**	**Mean ± SEM (median)**	**Mean ± SEM (median)**
immobile inside FA (%)	80.4 ± 0.5 (80)	80.2 ± 1.4 (80.3)	64.9 ± 2.3 (67.4)	81.6 ± 1.5 (80.8)	64.6 ± 1.4 (62.9)
confined inside FA (%)	13.1 ± 1.1 (14.7)	12.1 ± 0.7 (12)	17.1 ± 0.8 (16.3)	11.9 ± 0.8 (11.7)	15.4 ± 0.6 (14.4)
diffusive inside FA (%)	6.5 ± 0.8 (5.2)	7.7 ± 1.5 (6.9)	18 ± 1.6 (16.9)	6.5 ± 0.9 (5.7)	20 ± 1.4 (20.5)
immobile outside FA (%)	76.1 ± 2 (76.8)	74.5 ± 1.6 (76.1)	52.8 ± 2.8 (52.8)	73.2 ± 3.4 (74)	54.7 ± 3.1 (53.5)
confined outside FA (%)	15.2 ± 1.2 (13.9)	15.2 ± 1 (15.6)	20.2 ± 0.9 (20.5)	14.3 ± 1.5 (15.4)	18.5 ± 0.8 (17.6)
diffusive outside FA (%)	8.8 ± 1.2 (7.3)	10.3 ± 1.9 (8.9)	27 ± 2.1 (28)	12.6 ± 2.2 (10.2)	26.9 ± 2.5 (30.6)
D_{diff} inside x10^3 (µm^2.s^{-1})	105.5 ± 5.4 (64.4)	107.7 ± 3.4 (72.1)	96.5 ± 2.1 (58.2)	114.7 ± 4.6 (54.7)	110.7 ± 2.7 (59.6)
D_{diff} outside x10^3 (µm^2.s^{-1})	200.5 ± 7.9 (149.1)	169.9 ± 3.8 (121.3)	175.7 ± 3.9 (111.8)	120.6 ± 4.8 (75.3)	236.6 ± 5 (131.7)

	Kindlin-2-WT (Fig 3)	Kindlin-2-WT + Mn2+ activation	Kindlin-2-QW	Kindlin-2-WT (Fig S3,4)	Kindlin-2-L357A
cells	17	17	33	12	22
trajectories inside FA	24941	33799	38495	15959	39848
trajectories outside FA	10677	42414	17831	7926	16832
	**Mean ± SEM (median)**	**Mean ± SEM (median)**	**Mean ± SEM (median)**	**Mean ± SEM (median)**	**Mean ± SEM (median)**
immobile inside FA (%)	67.1 ± 2.3 (67.6)	65.8 ± 2 (63)	53.3 ± 1.8 (55.4)	60.8 ± 2.7 (61.2)	60.9 ± 2.1 (60.5)
confined inside FA (%)	14.6 ± 1.2 (12.8)	14.6 ± 0.7 (15)	19.8 ± 0.9 (18.7)	18.3 ± 1.2 (18.1)	18.8 ± 1 (18.1)
diffusive inside FA (%)	18.3 ± 1.3 (18.9)	19.6 ± 1.5 (20.7)	26.9 ± 1.2 (26.2)	20.9 ± 1.9 (19.5)	20.3 ± 1.3 (20.1)
immobile outside FA (%)	58.9 ± 2.5 (59.7)	58.1 ± 2.5 (57.2)	52.8 ± 2.6 (50.2)	49.9 ± 2.6 (49.5)	53.2 ± 2.8 (53)
confined outside FA (%)	17.1 ± 0.8 (17.3)	15.8 ± 0.9 (15.3)	19.7 ± 1 (21)	20.9 ± 1.3 (20.7)	20.8 ± 0.8 (20.7)
diffusive outside FA (%)	24 ± 2 (21.7)	26.1 ± 1.8 (26.2)	27.6 ± 1.9 (28.9)	29.2 ± 1.9 (28.9)	26 ± 2.1 (26.7)

D_{diff} inside x10[3] ($\mu m^2.s^{-1}$)	85.1 ± 1.7 (48.6)	92.1 ± 1.5 (50.9)	107.1 ± 1.3 (62.3)	101.7 ± 2 (58.9)	100 ± 1.2 (59.3)
D_{diff} outside x10[3] ($\mu m^2.s^{-1}$)	181.3 ± 4.4 (116.5)	190 ± 2.2 (114.7)	246.6 ± 4.0 (162.8)	215.9 ± 4.2 (142.6)	196 ± 2.9 (127.3)

	Kindlin-2-K390A	Kindlin-2-WT (fig 4, S7)	Kindlin-2-ΔPH (MEF)	Kindlin-2-ΔPH-CAAX	PH domain
cells	23	15	28	18	18
trajectories inside FA	39632	16955	11295	10926	5831
trajectories outside FA	18734	6891	6483	11829	10397
	**Mean ± SEM (median)**	**Mean ± SEM (median)**	**Mean ± SEM (median)**	**Mean ± SEM (median)**	**Mean ± SEM (median)**
immobile inside FA (%)	64.1 ± 2.1 (68)	63 ± 2.1 (64.3)	66.3 ± 2.1 (66.3)	52 ± 1.5 (50.5)	60.6 ± 2.8 (57.3)
confined inside FA (%)	17.3 ± 1 (14.9)	18.4 ± 0.9 (18.3)	21.6 ± 1.3 (21)	20.8 ± 0.5 (21.4)	17.4 ± 1 (17.8)
diffusive inside FA (%)	18.7 ± 1.2 (16.7)	18.6 ± 1.3 (18.5)	12.1 ± 1 (12.3)	27.2 ± 1.3 (27.3)	22 ± 2.1 (24.7)
immobile outside FA (%)	59.2 ± 1.7 (59.2)	48.8 ± 1.9 (49.6)	65.7 ± 1.7 (68.6)	28.7 ± 1.7 (28.5)	49.1 ± 4.3 (42.3)
confined outside FA (%)	19 ± 0.6 (18.7)	21.7 ± 1.1 (21)	20.8 ± 1 (19.8)	26.6 ± 0.8 (26.9)	19.2 ± 1.4 (18.9)
diffusive outside FA (%)	21.8 ± 1.4 (22.9)	29.5 ± 1.4 (29.9)	13.5 ± 0.9 (13)	44.7 ± 1.3 (45.1)	31.7 ± 3.1 (34.9)
D_{diff} inside x10[3] ($\mu m^2.s^{-1}$)	107.5 ± 1.5 (62.2)	108.5 ± 2.2 (68.1)	121.8 ± 3.9 (74.3)	146.2 ± 2.8 (97.6)	241.3 ± 9.2 (141.5)
D_{diff} outside x10[3] ($\mu m^2.s^{-1}$)	205.2 ± 3.5 (133.2)	209.6 ± 4.7 (146)	202.3 ± 7.4 (134)	319 ± 3.5 (260.4)	343.9 ± 6.6 (242)

	Kindlin-2-WT (fig S6)	Paxillin	Kindlin-2-WT (Kind^{Ko}, fig S8a-e)	Kindlin-2-ΔPH (Kind^{Ko})	Kindlin-2-WT (Kind^{Ko}, fig S8f,g)
cells	10	12	11	18	8
trajectories inside FA	22501	16678	14940	9318	14279
trajectories outside FA	11406	2193	15985	8634	6115
	**Mean ± SEM (median)**	**Mean ± SEM (median)**	**Mean ± SEM (median)**	**Mean ± SEM (median)**	**Mean ± SEM (median)**
immobile inside FA (%)	72.2 ± 1 (73)	78.5 ± 0.9 (78.9)	58.1 ± 2.4 (58.9)	58.9 ± 2 (59.7)	67.9 ± 2.7 (68.9)
confined inside FA (%)	13.2 ± 0.8 (12.9)	11.4 ± 0.9 (11.4)	20.5 ± 1.1 (19.1)	22.8 ± 1 (23)	13 ± 1.2 (12.1)
diffusive inside FA (%)	14.6 ± 0.8 (15.1)	10.1 ± 0.4 (10.2)	21.4 ± 2.5 (17.7)	18.2 ± 1.6 (18.5)	19.1 ± 1.6 (19.3)
immobile outside FA (%)	49.5 ± 1.8 (49.8)	77.6 ± 2 (79.9)	41.3 ± 3.9 (42.0)	59.5 ± 3.2 (62.0)	57.1 ± 3.1 (59.5)
confined outside FA (%)	19.8 ± 0.7 (20.3)	12.3 ± 1.7 (9.4)	23.6 ± 1.0 (24.3)	20.4 ± 1.8 (19.9)	15.6 ± 1 (14.7)
diffusive outside FA (%)	30.8 ± 1.8 (29.7)	10.1 ± 1 (10.4)	35.1 ± 3.4 (35.0)	20.1 ± 1.8 (17.8)	27.4 ± 2.2 (26.3)
D_{diff} inside x10[3] ($\mu m^2.s^{-1}$)	106 ± 3.1 (59.9)	87 ± 1.9 (53.8)	126.8 ± 2.4 (74)	102.7 ± 2.6 (63.8)	103 ± 2.8 (58.9)

D_{diff} outside x10^3 (µm^2.s^{-1})	182.9 ± 2.9 (130.1)	251.1 ± 23.2 (119.2)	275.7 ± 3.6 (176.3)	249.5 ± 6.7 (153.9)	270.1 ± 8.1 (160.3)

	Kindlin-2-WT + Mn2+ activation (Kind^{Ko})	Kindlin-2-QW (Kind^{Ko})	Kindlin-2-WT (Kind^{Ko}, fig S8h,i)	Kindlin-2-L357A (Kind^{Ko})
cells	4	4	6	4
trajectories inside FA	11114	2798	6095	3033
trajectories outside FA	9745	2535	2895	1297
	**Mean ± SEM (median)**	**Mean ± SEM (median)**	**Mean ± SEM (median)**	**Mean ± SEM (median)**
immobile inside FA (%)	63.6 ± 2.6 (63.4)	55.6 ± 4.6 (54.8)	59.7 ± 3.1 (63)	59.2 ± 3.2 (62)
confined inside FA (%)	14.6 ± 0.7 (14.7)	20 ± 2.6 (20.9)	23.2 ± 1 (23.9)	22.7 ± 1.3 (21.8)
diffusive inside FA (%)	21.8 ± 2.1 (22.2)	24.4 ± 2.2 (24.3)	17.1 ± 2.5 (15.6)	18.1 ± 2.1 (16.7)
immobile outside FA (%)	57.2 ± 6.6 (56.8)	55.4 ± 6.9 (53.6)	45.4 ± 4 (43.5)	50.5 ± 4.7 (50.9)
confined outside FA (%)	15.7 ± 1.7 (16.1)	21 ± 3.1 (20.6)	24.8 ± 0.8 (25.5)	25.4 ± 1.8 (24.8)
diffusive outside FA (%)	27.1 ± 4.9 (26.9)	23.6 ± 4.1 (24.8)	29.8 ± 3.2 (30.6)	24.1 ± 3.1 (23)
D_{diff} inside x10^3 (µm^2.s^{-1})	103.4 ± 2.4 (62.1)	90.2 ± 3.3 (58.3)	132.9 ± 4.7 (82.3)	135.7 ± 5.6 (92.6)
D_{diff} outside x10^3 (µm^2.s^{-1})	264.1 ± 5.9 (148.5)	242 ± 10.8 (138.7)	333.2 ± 10.3 (237.1)	289.9 ± 18 (195.4)

	Kindlin-2-WT (spreading MEFs fig S3)	Kindlin-2-QW (spreading MEFs fig S3)
cells	11	3
trajectories inside NA	21327	3065
trajectories outside NA	18052	2676
	**Mean ± SEM (median)**	**Mean ± SEM (median)**
immobile inside NA (%)	54.7 ± 1.8 (55.5)	38.5 ± 7.2 (38.4)
confined inside NA (%)	15.6 ± 1.2 (15.9)	21.5 ± 3.0 (22.6)
diffusive inside NA (%)	29.7 ± 0.9 (28.7)	40.0 ± 5.7 (35.6)
immobile outside NA (%)	51.1 ± 2.6 (47.8)	47.0 ± 2.0 (48.1)
confined outside NA (%)	16.5 ± 1.0 (17.0)	18.4 ± 1.5 (17.4)
diffusive outside NA (%)	32.4 ± 1.8 (31.7)	34.6 ± 3.0 (33.0)
D_{diff} inside x10^3 (µm^2.s^{-1})	153.4 ± 2.3 (89.6)	214.0 ± 6.7 (124.8)

D_{diff} outside $x10^3$ ($\mu m^2.s^{-1}$)	212.1 ± 2.8 (144.3)	259.3 ± 9.1 (173.9)

Table S2. Results of immobilization time measurements inside focal adhesions by PALM.

	β1-integrin-WT	β1-integrin-Y795A	β1-integrin-Y783A	β3-integrin-WT	β3-integrin-Y759A	β3-integrin-Y747A
cells	3	6	6	6	5	5
immobilizations	296	229	272	557	496	464
Immobilization time (mean ± SEM (median), seconds)	22.29 ± 1.62 (12)	10.81 ± 1.12 (5)	15.28 ± 1.72 (5.25)	40.99 ± 1.93 (25)	32.97 ± 1.85 (20)	31.05 ± 1.79 (19)

	Kindlin-2-WT	Talin-1
cells	12	7
immobilizations	1127	584
Immobilization time (mean ± SEM (median), seconds)	11.89 ± 0.38 (7.5)	22.48 ± 0.90 (16)

Table S3. Results of membrane fraction experiments.

	Kindlin-2-WT (MEF)	Kindlin-2-ΔPH (MEF)	Kindlin-2-QW	Kindlin-2-K390A	Kindlin-2-L357A
cells	28	27	15	15	15
Membrane fraction (Mean ± SEM (median), arbitrary unit)	1.41 ± 0.05 (1.35)	0.64 ± 0.02 (0.62)	1.05 ± 0.03 (1.03)	1.24 ± 0.05 (1.25)	1.23 ± 0.05 (1.24)

	β3-integrin-WT	mEos2	Kindlin-2-WT ($Kind^{Ko}$)	Kindlin-2-ΔPH ($Kind^{Ko}$)	Kindlin-2-ΔPH-CAAX
cells	17	13	21	20	21
Membrane fraction (AU, mean ± SEM (median))	1.32 ± 0.04 (1.31)	0.56 ± 0.02 (0.53)	1.35 ± 0.07 (1.33)	0.33 ± 0.03 (0.28)	1.31 ± 0.06 (1.25)

Table S4. Results of DONALD experiments. Molecules detected above 200 nm were considered as non-specific and were excluded.

	Paxillin	Kindlin-2-WT	Kindlin-2-QW	Kindlin-2-ΔPH	Kindlin-2-ΔPH-CAAX
cells	6	6	9	7	7
detections inside FAs	2720922	2112741	2407581	2572448	3257051
detections outside FAs	1779915	2574857	2256572	2023314	4483186
z_{median} (mean ± SEM (median), nm) inside FAs	73.1 ± 0.03 (67.6)	68.1 ± 0.04 (63.0)	71.8 ± 0.03 (66.9)	82.7 ± 0.03 (78.7)	60.3 ± 0.03 (53.3)
z_{median} (mean ± SEM (median), nm) outside FAs	96.4 ± 0.04 (94.9)	84.6 ± 0.03 (81.5)	82.6 ± 0.04 (78.9)	93.0 ± 0.04 (90.7)	78.7 ± 0.03 (73.8)
Z_{peak} (mean ± SEM, nm) inside FAs	58.9 ± 4.3	48.7 ± 4.8	53.3 ± 2.7	66.3 ± 2.8	39.0 ± 0.7
Z_{peak} (mean ± SEM, nm) outside FAs	90.3 ± 5.0	69.2 ± 2.2	67.8 ± 3.5	81.9 ± 3.9	60.7 ± 2.5

Table S5. Results of focal adhesion (FA) enrichment experiments.

	Kindlin-2-WT	mEos2	Kindlin-2-ΔPH	Kindlin-2-QW	Kindlin-2-ΔPH-CAAX
cells	36	38	27	24	50
FA enrichment (Mean ± SEM (median), arbitrary unit)	2.17 ± 0.08 (2.09)	0.98 ± 0.03 (0.92)	1.17 ± 0.03 (1.19)	1.28 ± 0.02 (1.26)	1.77 ± 0.05 (1.64)

	Kindlin-2-L357A	Kindlin-2-K390A
cells	34	30
FA enrichment (Mean ± SEM (median), arbitrary unit)	1.78 ± 0.06 (1.76)	2.04 ± 0.08 (2.02)

Table S6. Results of cell spreading experiments. Percentages are expressed in the format "mean ± SEM (median)".

	Paxillin	Kindlin-2-WT	Kindlin-2-QW	Kindlin-2-ΔPH	Kindlin-2-ΔPH-CAAX	Kindlin-2-WT-CAAX
cells	141	312	284	311	309	289
non-spread (%)	96.2 ± 3 (98.3)	21.8 ± 4.6 (23)	35.4 ± 6.9 (41.8)	72.6 ± 7.3 (78.8)	43.5 ± 3.1 (44.6)	27.3 ± 7.9 (21.8)
partially spread (%)	3.8 ± 3 (1.7)	57.7 ± 1.7 (58.9)	56 ± 4.4 (52.4)	24.6 ± 6.4 (19.2)	47.8 ± 4.3 (45.5)	54.1 ± 1.9 (53.5)
spread (%)	0 ± 0 (0)	20.5 ± 6.1 (17)	8.5 ± 2.7 (7.1)	2.9 ± 0.9 (2)	8.7 ± 1.3 (9.9)	18.6 ± 6.3 (24.8)

Table S7. Results of focal adhesion experiments. Except cell numbers, values are expressed in the format "mean ± SEM (median)". FA: Focal adhesion.

	Kindlin-2-WT	Kindlin-2- ΔPH	Kindlin-2- ΔPH-CAAX	Kindlin-2-QW	Kindlin-2-WT-CAAX
cells	84	74	62	30	63
FA total area (μm²)	44.4 ± 2.9 (35.0)	10.7 ± 1.0 (8.8)	22.9 ± 1.3 (19.9)	22.4 ± 2.8 (18.0)	34.6 ± 1.9 (30.6)
FA count	58.0 ± 3.1 (52)	10.8 ± 1.1 (8.5)	25.7 ± 1.4 (24)	23.3 ± 2.3 (19)	41.9 ± 2.3 (41)
Average adhesion size / cell (μm²)	0.838 ± 0.060 (0.73)	1.071 ± 0.067 (0.994)	0.953 ± 0.057 (0.786)	1.093 ± 0.120 (0.948)	0.948 ± 0.056 (0.884)
cell area (μm²)	998.3 ± 49.3 (877.7)<br>90 cells	469.4 ± 41.5 (407.8)<br>76 cells	523.2 ± 35.6 (463.8)<br>68 cells	754.6 ± 77.9 (568.8)<br>32 cells	916.0 ± 61.4 (814.2)<br>63 cells
