# Supplementary material for: Molecular motion and tridimensional nanoscale localization of kindlin control integrin activation in focal adhesions
Source: Nat Commun. 2021 May 25;12:3104. doi: 10.1038/s41467-021-23372-w (PMC8149821; doi:10.1038/s41467-021-23372-w)
Supplement: Supplementary file 3 — Description of Additional Supplementary Files [file 41467_2021_23372_MOESM3_ESM.docx]

**Description of Additional Supplementary Files**

File Name: Supplementary Movie 1.Kindlin-2 undergoes free diffusion along the plasma membrane inside and outside FAs contrary to talin.

Description: This movie shows the time course of single protein tracking experiments depicted in Fig. 2. It covers a period of about 20 seconds, during which single kindlin-2-WT (left) and talin (right) proteins labeled by mEos2 are tracked inside and outside FAs (revealed by GFP-paxillin and outlined by blue lines). Scale bar = 1.6 μm. Speed, 0.6X.

File Name: Supplementary Movie 2.

Description: Deletion of the PH domain (kindlin-2-ΔPH) strongly inhibits membrane free diffusion both inside and outside FAs.

This movie shows the time course of the single protein tracking experiment depicted in Fig. 4. It covers a period of about 20 seconds, during which single kindlin-2-ΔPHproteins labeled by mEos2 are tracked inside and outside FAs (revealed by GFP-paxillin and outlined by blue lines). Scale bar = 1.6 μm. Speed, 0.6X.

File Name: Supplementary Movie 3. Restoring membrane association of kindlin-2-ΔPH by adding a CAAX prenylationsequence induces membrane recruitment and diffusion inside and outside FAs.

Description: This movie shows the time course of the single protein tracking experiment depicted in Supplementary Fig. S6. It covers a period of about 20 seconds, during which single kindlin-2-ΔPH-CAAXproteins labeled by mEos2 are tracked inside and outside FAs (revealed by GFP-paxillin and outlined by blue

lines). Scale bar = 1.6 μm. Speed, 0.6X.
