# Supplementary material for: Molecular motion and tridimensional nanoscale localization of kindlin control integrin activation in focal adhesions
Source: Nat Commun. 2021 May 25;12:3104. doi: 10.1038/s41467-021-23372-w (PMC8149821; doi:10.1038/s41467-021-23372-w)
Supplement: Supplementary file 7 — Reporting Summary [file 41467_2021_23372_MOESM7_ESM.pdf]

natureresearch

Corresponding author(s): Olivier Rossier & Grégory Giannone

Last updated by author(s): Mar 11, 2021

Reporting Summary

Nature Research wishes to improve the reproducibility of the work that we publish. This form provides structure for consistency and transparency in reporting. For further information on Nature Research policies, see Authors & Referees and the Editorial Policy Checklist .

Statistics

For all statistical analyses, confirm that the following items are present in the figure legend, table legend, main text, or Methods section.

n/a	Confirmed	
☐	☒	The exact sample size (n) for each experimental group/condition, given as a discrete number and unit of measurement
☐	☒	A statement on whether measurements were taken from distinct samples or whether the same sample was measured repeatedly
☐	☒	The statistical test(s) used AND whether they are one- or two-sided<br>*Only common tests should be described solely by name; describe more complex techniques in the Methods section.*
☒	☐	A description of all covariates tested
☐	☒	A description of any assumptions or corrections, such as tests of normality and adjustment for multiple comparisons
☐	☒	A full description of the statistical parameters including central tendency (e.g. means) or other basic estimates (e.g. regression coefficient) AND variation (e.g. standard deviation) or associated estimates of uncertainty (e.g. confidence intervals)
☐	☒	For null hypothesis testing, the test statistic (e.g. F, t, r) with confidence intervals, effect sizes, degrees of freedom and P value noted<br>*Give P values as exact values whenever suitable.*
☒	☐	For Bayesian analysis, information on the choice of priors and Markov chain Monte Carlo settings
☒	☐	For hierarchical and complex designs, identification of the appropriate level for tests and full reporting of outcomes
☒	☐	Estimates of effect sizes (e.g. Cohen's d, Pearson's r), indicating how they were calculated

Our web collection on statistics for biologists contains articles on many of the points above.

Software and code

Policy information about availability of computer code

Data collection	Image acquisitions were steered by the Metamorph software (Molecular Devices, version 7.10.2) as detailed in the methods section.
Data analysis	Single protein tracking (SPT) acquisitions were analyzed using the Metamorph software (Molecular Devices, version 7.10.2) using a custom computer code written for Metamorph (J.B. Sibarita, IINS, Bordeaux, France) fully described previously (Izzedin et al., Optics express 2012; Racine et al., IEE 2006 and Racine et al., Journal of Microscopy 2007) and available upon request at the following address [https://www.iins.u-bordeaux.fr/SIBARITA]. Full analysis of trajectories (diffusion coefficient D; distributions of D; diffusion modes (immobile, confined, free-diffusive); confinement radius) were performed with a custom routine written for Matlab using computation of the mean squared displacement as described previously (Rossier et al., Nature Cell Biology 2012; Chazeau et al., EMBO J 2014; Mehidi et al., Current Biol 2019). As stated in the code availability section, MATLAB (version 2007a) codes for analysis of sptPALM trajectories and MSD computing are available from the corresponding author on reasonable request. Kymographs were generated and analyzed using a custom ImageJ (version 1.53c) plugin (Kymo ToolBox, F. Cordelières, Bordeaux Imaging Center, BIC). Single molecule axial localization by direct optical nanoscopy with axially localized detection (DONALD) were analyzed using the commercially available Neo software (Abbelight, version 26) that was described elsewhere (Bourg et al., Nature Photonics 2015). Western blot membranes were revealed on a Chemidoc setup with Image Lab Software. Western Blot quantifications were obtained using ImageJ. For statistical analysis of the data, we used GraphPad Prism (version 8.4.3).

For manuscripts utilizing custom algorithms or software that are central to the research but not yet described in published literature, software must be made available to editors/reviewers. We strongly encourage code deposition in a community repository (e.g. GitHub). See the Nature Research guidelines for submitting code & software for further information.

Data

Policy information about availability of data

All manuscripts must include a data availability statement. This statement should provide the following information, where applicable:

- Accession codes, unique identifiers, or web links for publicly available datasets
- A list of figures that have associated raw data
- A description of any restrictions on data availability

The datasets generated during and/or analyzed during the current study are not publicly available due to their large size but are available from the corresponding authors on reasonable request.

Field-specific reporting

Please select the one below that is the best fit for your research. If you are not sure, read the appropriate sections before making your selection.

☒ Life sciences ☐ Behavioural & social sciences ☐ Ecological, evolutionary & environmental sciences

For a reference copy of the document with all sections, see nature.com/documents/nr-reporting-summary-flat.pdf

Life sciences study design

All studies must disclose on these points even when the disclosure is negative.

Sample size

No statistical calculations were used to predict the sample size. All results for each condition correspond to datasets from at least 2 independent experiments excepted for DONALD experiments where shown data correspond to a single experiment providing more than 10e6 single molecule localizations per condition. Arithmetic means, median and s.e.m. were calculated and are shown in graphs. Respective n values are shown in figure captions and Supplementary Tables 1-7.

For SPT experiments, the sample size was not pre-chosen, but we imaged from 3 to 33 cells per condition to account for the differences of diffusive behavior and differences of density of detection and trajectories due to cell to cell variability. Each cell was analyzed independently and the distribution of the diffusion coefficients computed from 4000-100,000 trajectories. The statistical n for the fractions of trajectories in the different diffusion modes (immobile, confined, free-diffusive) corresponds to the number of cell analyzed (Fig. 1-4; Supplementary Fig. 2-8; Supplementary Table 1). Statistical n for diffusion coefficient correspond to the number of trajectories (Fig. 2-4; Supplementary Fig. 1-8; Supplementary Table 1). For kymograph analyses of mEos2-tagged proteins immobilization time inside FAs generated from sptPALM super-resolution time-lapse sequences (2 Hz), we imaged from 3 to 12 cells, generating between 229 to 1127 immobilization events per condition to account for cell to cell variability (Fig. 1-2; Supplementary Table 2). Statistical n for immobilization time correspond to the number of events (Fig. 1-2; Supplementary Table 2). For assessing the fraction of membrane associated-protein we imaged from 13 to 28 cells per condition to account for cell to cell variability. Statistical n for membrane fraction correspond to the number of cell analyzed (Fig. 4; Supplementary Fig. 7; Supplementary Table 3). For DONALD experiments, we imaged from 6 to 9 cells per condition to account for cell to cell variability. Each cell was analyzed independently and the distribution of single molecule axial localizations of proteins computed from 1.7x10e6-4.5x10e6 single molecule detections. Statistical n for single molecule axial localization correspond to the number of single molecule detections (Fig. 5; Supplementary Table 4). To determine FA enrichment of proteins, we imaged from 24 to 50 cells per condition to account for cell to cell variability. Statistical n for FA enrichment correspond to the number of cell analyzed (Fig. 6; Supplementary Table 5). For cell spreading rescue experiments with the kindlin dKO cell line, we imaged from 141 to 312 cells per condition to account for cell to cell variability in order to determine the relative fraction of non-spread, partially spread, and spread kindlin dKO cells after re-expression of kindlin-2-WT or mutated variants averaged from 3 independent experiments (Fig. 7; Supplementary Table 6). For FA rescue experiments with the kindlin dKO cell line, we imaged from 30 to 84 cells per condition to account for cell to cell. Unless specified as for cell area, statistical n for total FA area per cell, number of FA per cell, average FA size per cell correspond to the number of cell analyzed (Fig. 7; Supplementary Fig. 9; Supplementary Table 7). Statistical significances were obtained using a non-parametric, two-tailed Mann–Whitney rank sum test for all the parameters decribed above. The indicated P values were obtained with the two-tailed, non-parametric Mann–Whitney rank sum test using the software GraphPad Prism version 8.4.3, except for z distributions where the Mann–Whitney rank sum test was performed using Matlab 2007a due to very high sample size. The exact P values are indicated on all the main and supplementary figures except when $P < 0.0001$.

Data exclusions

For DONALD experiments, every single molecules detections were included for the Z distributions (Fig. 5f,g). For the box plot (Fig. 5h; Supplementary Table 4), molecules detected above 200 nm were discarded to improve the relevance of the displayed median and average z positions (pre-established criteria as DONALD modality axial localization precision decreases strongly above 150-200 nm from the coverslip).

In the image display (Fig.5a-e), single molecules detected 150 nm above the surface were considered as nonspecific and were discarded (criteria not pre-established) to improve the contrast in the focal adhesions/plasma membrane layer. For ease of observation, the obtained images were smoothed using a xy mean filter with a 5x5 kernel. For cell spreading rescue experiments with the kindlin dKO cell line, non-isolated cells (i.e., in contact with other cells) and polynucleated cells were excluded in a pre-established manner from the analysis of phase contrast images (Fig. 7a,b) to ensure no interference from cell-cell contacts or cell deviation in culture conditions. As transfection efficiency was checked with the GFP-paxillin signal to ensure that re-expression of kindlin-2-WT and mutants was achieved, cells showing a GFP-paxillin average signal below 1.5 fold of the average background fluorescence signal were also excluded (this criteria was not pre-established). For FA rescue experiments with the kindlin dKO cell line, non-isolated cells (i.e., in contact with other cells) and polynucleated cells were excluded (pre-established criteria) from the analysis of GFP-paxillin images (Fig. 7c-e; Supplementary Fig. 9).

Replication

All results for each condition correspond to data from at least 3 independent experiments with identical results unless indicated in the figure legends. This is detailed in the 'Sample Size' section, along with description of statistical n for each datasets. All experimental conditions were carefully normalized to minimize variability. This includes, cell transfection, delay between transfection and imaging, cell density.

Randomization	All analyzed cells were selected for their expression level of fluorescently-tagged proteins, hence no randomization was applied to the following study.
Blinding	Our experiments were not done blind. However, we used computer-based single molecule detection and tracking and analysis with as many cells as was practical to analyse to minimise bias.

Reporting for specific materials, systems and methods

We require information from authors about some types of materials, experimental systems and methods used in many studies. Here, indicate whether each material, system or method listed is relevant to your study. If you are not sure if a list item applies to your research, read the appropriate section before selecting a response.

Materials & experimental systems

n/a	Involved in the study
☐	☒ Antibodies
☐	☒ Eukaryotic cell lines
☒	☐ Palaeontology
☒	☐ Animals and other organisms
☒	☐ Human research participants
☒	☐ Clinical data

Methods

n/a	Involved in the study
☒	☐ ChIP-seq
☒	☐ Flow cytometry
☒	☐ MRI-based neuroimaging

Antibodies

Antibodies used	Home-made anti-GFP nanobody labelled with AlexaFluor-647; Monoclonal anti-kindlin-2 antibody (Merck, Cat# MAB2617, Clone 3A3, Lot# 3114519); Monoclonal anti-β-actin antibody (Sigma-Aldrich, Cat# A5316, Clone AC-74).
Validation	The home-made anti-GFP nanobody labelled with AlexaFluor-647 was validated in 'Chamma et al. Optimized labeling of membrane proteins for applications to super-resolution imaging in confined cellular environments using monomeric streptavidin. Nat. Protoc. 12, 748–763 (2017)'. Validation statements for commercial anti-kindlin-2 and anti-β-actin antibodies can be found on the suppliers' website catalog. For anti-kindlin-2 antibody, quality assurance was evaluated by western blot analysis in HeLa cell lysate (0.5 µg/ml of this antibody detected Kindlin-2 in 10 µg of HeLa cell lysate - https://www.merckmillipore.com/FR/fr/product/Anti-Kindlin-2-Antibody-clone-3A3,MM_NF-MAB2617#overview). For anti-β-actin antibody, quality assurance was evaluated by western blot analysis of whole cell extract of human foreskin fibroblasts separated on SDS-PAGE (https://www.sigmaaldrich.com/content/dam/sigma-aldrich/docs/Sigma/General_Information/1/a5316blot.pdf).

Eukaryotic cell lines

Policy information about cell lines

Cell line source(s)	The mouse embryonic fibroblast cell line used in this study was previously described in: Su J, Muranjan M, Sap J. (1999). Receptor protein tyrosine phosphatase alpha activates Src-family kinases and controls integrin-mediated responses in fibroblasts. Curr Biol. 9(10):505-11. Primary embryonic fibroblasts were isolated from E13–E15 day old RPTPα+/+ embryos. A retroviral vector expressing polyoma large T antigen from the SV40 virus was used to immortalize the cultures.<br><br>The KindKo cell line was previously described in: Theodosiou, M. et al. Kindlin-2 cooperates with talin to activate integrins and induces cell spreading by directly binding paxillin. Elife 5, 1–24 (2016). Mouse fibroblasts were derived from the kidneys of 21 d old mice, immortalized by retrovirally transducing the SV40 large T antigen, cloned (KindCtr) and finally infected with an adenovirus to transduce the Cre recombinase resulting in kindlin-null (KindKo) cells.
Authentication	Authentication was performed in the studies describing the generation of these 2 cell lines. The authentication for mouse embryonic fibroblasts (MEFs) and mouse kidney fibroblasts (KindKo) cell lines was performed by the laboratory responsible for the cell line generation. For KindKo cells, they are barely adherent in culture conditions unless kindlin-2 is reintroduced by DNA plasmid transfection.
Mycoplasma contamination	The cell line used in this study is regularly tested for Mycoplasma, all cells lines have been verified as Mycoplasma free.
Commonly misidentified lines (See ICLAC register)	The cell line used in this study is not a misidentified line.
